# Investigation of the Young’s Modulus and the Residual Stress of 4H-SiC Circular Membranes on 4H-SiC Substrates

**DOI:** 10.3390/mi10120801

**Published:** 2019-11-21

**Authors:** Jaweb Ben Messaoud, Jean-François Michaud, Dominique Certon, Massimo Camarda, Nicolò Piluso, Laurent Colin, Flavien Barcella, Daniel Alquier

**Affiliations:** 1 GREMAN UMR-CNRS 7347, Université de Tours, INSA Centre Val de Loire, 37071 Tours, France; jaweb.benmessaoud@univ-tours.fr (J.B.M.); jean-francois.michaud@univ-tours.fr (J.-F.M.); dominique.certon@univ-tours.fr (D.C.); laurent.colin@univ-tours.fr (L.C.); flavien.barcella@univ-tours.fr (F.B.); 2 Paul Scherrer Institute, ODRA/116, 5232 Villigen, Switzerland; massimo.camarda@psi.ch; 3 STMicroelectronics, Stradale Primosole, 50, 95121 Catania, Italy; nicolo.piluso@st.com

**Keywords:** 4H-SiC, bulk micromachining, electrochemical etching, circular membrane, bulge test, vibrometry, mechanical properties, Young’s modulus, residual stress, FEM

## Abstract

The stress state is a crucial parameter for the design of innovative microelectromechanical systems based on silicon carbide (SiC) material. Hence, mechanical properties of such structures highly depend on the fabrication process. Despite significant progresses in thin-film growth and fabrication process, monitoring the strain of the suspended SiC thin-films is still challenging. However, 3C-SiC membranes on silicon (Si) substrates have been demonstrated, but due to the low quality of the SiC/Si heteroepitaxy, high levels of residual strains were always observed. In order to achieve promising self-standing films with low residual stress, an alternative micromachining technique based on electrochemical etching of high quality homoepitaxy 4H-SiC layers was evaluated. This work is dedicated to the determination of their mechanical properties and more specifically, to the characterization of a 4H-SiC freestanding film with a circular shape. An inverse problem method was implemented, where experimental results obtained from bulge test are fitted with theoretical static load-deflection curves of the stressed membrane. To assess data validity, the dynamic behavior of the membrane was also investigated: Experimentally, by means of laser Doppler vibrometry (LDV) and theoretically, by means of finite element computations. The two methods provided very similar results since one obtained a Young’s modulus of 410 GPa and a residual stress value of 41 MPa from bulge test against 400 GPa and 30 MPa for the LDV analysis. The determined Young’s modulus is in good agreement with literature values. Moreover, residual stress values demonstrate that the fabrication of low-stressed SiC films is achievable thanks to the micromachining process developed.

## 1. Introduction

Silicon carbide (SiC) is recognized as an excellent material for power applications due to notable electrical properties but SiC is also an outstanding candidate for microelectromechanical systems (MEMS) thanks to its distinguished chemical and mechanical properties [1]. Therefore, sensors that are able to detect temperature, gas, and pressure in aerospace or the automotive field are potential applications of SiC-based devices [2,3,4]. The cubic 3C-SiC polytype is favoured for MEMS applications as it can be epitaxially grown on silicon (Si) substrates and thus offers a low-cost solution for SiC-based MEMS development coupled with the conventional Si technologies. Unfortunately, the lattice mismatch and thermal expansion coefficient difference between the epitaxied 3C-SiC film and the Si substrate lead to high residual stress after deposition and cooling steps [5]. Generally, 3C-SiC suspended films exhibit an important level of stress, typically more than 100 MPa which consequently affects the film mechanical reliability. In order to overcome the difficulties related to the heteroepitaxy, an alternative solution to achieve suspended films, based on SiC, is using the electrochemical etching (ECE) of 4H-SiC wafers. Until now, very few papers deal with the fabrication of 4H-SiC MEMS structures. Thus, Nida et al. proposed a promising technique employing a highly selective etching of 4H-SiC homoepitaxy films [6]. The etching process stops at the interface between n^+^ 4H-SiC substrate and n^−^ 4H-SiC epilayer, enabling the fabrication of freestanding thin films. Moreover, the crystalline quality of the epilayer, and so, the suspended thin-film, is not affected by the process [7]. This method could pave the road for the fabrication of novel SiC-based detectors [6]. However, designing such original film for MEMS applications requires knowing the mechanical properties of the epilayers. Several popular approaches exist to monitor the static behavior of free-standing films: Curvature measurements [8,9], beam-bending testing [10,11], Raman spectroscopy [12,13], nanoindentation [14], and bulge test [15,16]. Moreover, dynamic techniques based on the resonance frequency determination of thin film are also intensively used [17,18]. In order to determinate the mechanical properties (Young’s modulus and residual stress values) of the 4H-SiC film, this study aims to evaluate two experimental techniques: The bulge test and the vibrating method.

The static deflection analysis of a circular shape membrane submitted to high external pressure (up to 4 bars) was implemented. In other words, this method enables to discriminate easily residual stress effects from plate behavior and so, to measure simultaneously residual stress and Young’s modulus values. In addition, the static load-deflection curve of a circular film is described by a simple analytical expression, that depends on two fitting parameters only, easy to determine from experimental data [19]. However, several relations between these fitting parameters and the mechanical properties of the film were proposed in the literature, leading to a non-unique solution for the mechanical property value determination [20]. Therefore, a complementary method, based on the membrane vibration study, was used to help in the determination of the thin-film mechanical properties. For that, the dynamic behavior of the film was measured by means of laser Doppler vibrometry (LDV), i.e., resonance mode investigations. An inverse problem approach, based on finite element computations, was also implemented to determine the mechanical properties of the fabricated film.

This study focused on the characterization of a circular 4H-SiC freestanding film. After the membrane preparation description, the methods and experiments are presented. The Young’s modulus and the residual stress of the membrane are then extracted using the bulge test results. Finally, combining the resonance frequency measurements with the finite element model allowed refining the residual stress value. This last parameter seems to have an important influence on the membrane mechanical behavior.

## 2. Materials and Methods

### 2.1. Sample Preparation

The 4H-SiC membrane was fabricated starting from a wafer with homo-epitaxial layer grown on 375 µm thick 10^18^ cm^−3^ n-type substrate (supplied from CREE^®^, Durham, NC, USA). The epitaxial layer is around 9 µm thick 10^13^ cm^−3^ n-type. The substrate removal was obtained by electrochemical etching. ECE is an oxidation/oxide-removal process obtained by dipping the SiC wafer in a hydrofluoridric acid-based solution and electrically supply holes for the oxidation through a 100 nm aluminium back metal contact [6,21,22,23]. The process is capable of removing highly doped (≥ 10^18^ cm^−3^) p-type and n-type layers but is selective towards low-doped n-type layers (selectivity > 1000:1 with respect to the 5 × 10^13^ cm^−3^ doped n^−^ layer). Hence, this process allows the full removal of the highly doped substrate and the local release of the epitaxial layers. The realized membranes have thicknesses and uniformities determined by the epitaxial layer. The 4H-SiC suspended film with circular shape was fabricated at the Paul Scherrer Institute [6]. In addition, the circular shape is the most appropriate geometry in order to study the effects related to the internal stress of a film. Indeed, the stress is equi-biaxial, so the loading will not produce any discontinuity.

**Figure 1:**
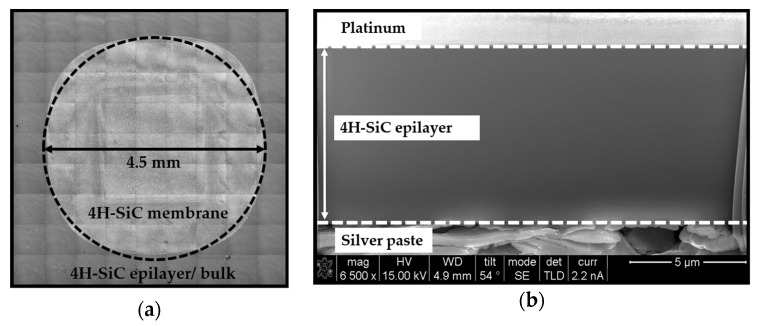
(**a**) Laser Scanning Microscope (LSM) image of the 4H-SiC membrane using stitching mode, obtained after the electrochemical etching (ECE) process. Circular insert added to show that the 4H-SiC membrane can be assimilated as a circle. (**b**) Focused ion beam (FIB) cross-section image allowing the membrane thickness determination.

A Lext OLS4100 Laser Scanning Microscope (LSM), from Olympus Corporation (Shinjuku-ku, Tokyo, Japan), was used in order to observe the full membrane using the stitching mode. Figure 1a shows the image of the membrane shape after ECE process. One can observe a slight membrane asymmetric geometry, which is due to the fabrication process, i.e., to a non-uniform under-etching of the substrate with respect to the etching mask. Therefore, we assumed that the membrane can be reasonably assimilated as a circle. The diameter measurements were performed in several directions and an average diameter value was evaluated at 4.5 mm. Thickness determination was performed using a Strata DB235 Focused Ion Beam (FIB), from Thermo Fisher Scientific (Waltham, MA, USA). To do that, we injected silver paste through the cavity of the membrane in order to fix and stiffen the suspended film to prevent any vibrations during the observations. After that, the FIB cross-section images were carried out in order to obtain a direct measurement of the film thickness as shown in Figure 1b. Thus, a membrane thickness of 8.8 µm was measured and a thickness variation of ± 0.2 µm was evaluated. As expected, the thin film has a well-controlled thickness because the 4H-SiC epilayer acts as an etching stop.

### 2.2. Circular Membrane Deflection under Uniform Pressure

**Figure 2:**
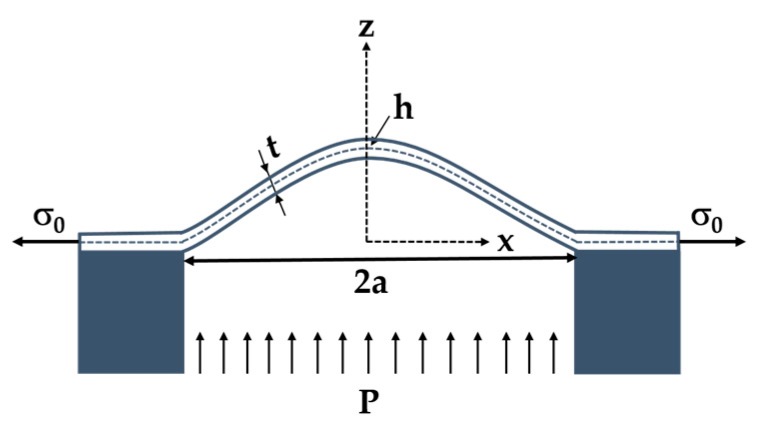
Schematic cross-sectional membrane with initial in-plane tension under uniform pressure *P*.

The bulge test method consists in submitting a membrane to uniform external pressure in order to observe its load-deflection behavior. At low value, the mechanical return forces of the membrane are mainly due to the residual stress. Whereas, when the pressure value increases, the return forces from plate stiffness govern the displacement. The deflection of a clamped membrane is measured according to the applied pressure. The most common pressure-deflection relationship of a pre-stressed circular membrane can be expressed as [24]:

(1) $$P\left(h\right)=\;C_{1}\frac{t\sigma_{0}}{a^{2}}h+C_{2}\frac{t}{a^{4}}\left(\frac{E}{1-v}\right)h^{3}=\;Ah+Bh{,}^{3}$$

where *P* is the applied pressure, *t* is the membrane thickness, *σ*_0_ is the residual stress, *a* is the membrane radius, *h* is the maximum bulge deflection at the centre of the membrane, *E* is the Young’s modulus, and *υ* is the Poisson’s ratio of the 4H-SiC thin-film. *C_1_* and *C_2_* are dimensionless constants, which depend on the membrane shape. Note that *C_2_* is also a function of the Poisson’s ratio. A schematic representation of a circular diaphragm is shown in Figure 2. By fitting the applied pressure as a function of the measured deflection, *A* and *B* coefficients can be estimated, leading to the determination of the Young’s modulus and the residual stress.

The models describing the load-deflection behavior of a circular plate as a function of the pressure have been extensively discussed. Several authors examined the large deflection behavior for the pure plate case, originally described by Nádai and Way [25,26]. Beams was the first to report an experimental model using bulge test to measure the mechanical properties of thin films deposited on substrates [27]. For a circular membrane, Beams determined *C_1_* and *C_2_* values, 4 and 8/3, respectively. A more accurate numerical solution indicated that *C_2_* can be expressed as (8/3) × (1.015 − 0.247*υ*). Table 1 summarizes the reported value of *C_1_* and *C_2_* for circular suspended films from literature. For comparison purposes, *C_2_* values assuming *υ* = 0.25 are also listed.

### 2.3. Membrane Vibration

#### 2.3.1. Analytical Description

The natural frequencies and mode shapes of membranes are a function of the structural properties and boundary conditions. Let us consider a circular membrane with a uniform thickness *t* and firmly clamped at its periphery. If the displacement of the membrane along the *z*-axis is small compared to the diameter, the stress state is isotropic and the effect of the fluid (air) is negligible [19]. Therefore, in small deflection case, an ideal membrane is described by assuming that the residual stress is higher than the bending rigidity. Thus, the membrane can support only tensile loads [33]. The mode shapes are described by two spatial coordinates and identified by the nodal line (m, n). Here, m and n are the index relative to the modal diameter lines and the number of nodal circle lines, respectively [33]. The natural frequencies of vibration for a circular membrane can be described as:

(2) $$f_{{\left(m,\;n\right)}_{\;}}=\;\frac{\alpha_{mn}}{2\pi a}\frac{\sqrt{\sigma_{0}}}{\rho },$$

where *a* is the membrane radius, *σ*_0_ is the residual stress, *ρ* is the density of the 4H-SiC thin-film and *α_mn_* values are derived from the roots of the first order Bessel functions. Zeros of the Bessel functions can be computed and tabulated [17].

#### 2.3.2. Finite Element Method Approach

A behavioral model of the membrane, based on the finite element method (FEM), was implemented to theoretically determine the mechanical properties of the fabricated film. Therefore, FEM calculations were used as virtual experiments to determine the natural frequencies and mode shapes of the membrane. The numerical model was built with the commercial COMSOL Multiphysics software package using eigenfrequency and solids mechanics modules. In the simulation procedure, a three-dimensional pre-stressed circular membrane was created. Perfectly clamped boundary conditions were used corresponding to a null mechanical displacement at the outer edge, in *x, y,* and *z* directions. The mesh settings were defined using the physics-controlled mesh option proposed by COMSOL Multiphysics, giving hence the following parameters: Tetrahedral shape elements and 8767 meshing nodes. These parameters were validated by comparing theoretical resonance frequency values of circular membranes to values obtained from FEM, a difference lower than 1% was obtained.

The residual stress and Young’s modulus values extracted from the bulge test measurements were used as input parameters.

### 2.4. Load-Deflection Measurements

**Figure 3:**
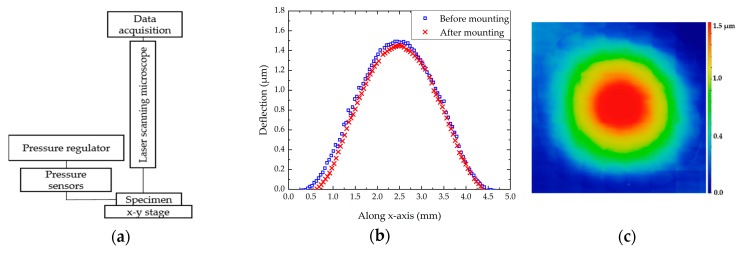
(**a**) Schematic of bulge test apparatus; (**b**) deflection of the circular 4H-SiC membrane, before and after, sample mounting; (**c**) typical topography used to measure the diaphragm deflection with LSM measurement.

The bulge test setup is fully discussed in the literature, mainly for improving the methodology and the technique accuracy [16,29,34]. Indeed, the deflection measurement errors can lead to an over or under estimation of the mechanical properties. Consequently, this method requires paying special attention to the chip preparation and deflection measurements. The experimental apparatus is schematically presented in Figure 3a. The sample was mounted on a 3 × 3 cm^2^ printed circuit board (PCB) holder with a drilled hole in the centre. Several studies have highlighted the importance of the bonding step for the reliability of the deflection measurements. The most common approach to fix the sample is to add adhesive around its edges. In such case, Jayaraman et al. observed that the sample moved during the measurement for a pressure up to 2.8 bars [35]. Mitchell et al. proposed a multi-step bonding method to seal and constrain the sample to the chuck without any displacement of the substrate [34]. Inspired by this method, we deposited an Ablebond 84-3J epoxy adhesive on the PCB and mounted the sample on it. Then, we applied the adhesive around all the edges of the sample to seal and prevent air leakage. Lastly, an annealing step of 1 h at 150 °C was carried out. For the bulge testing, the chip was placed in an airtight square cell, drilled on two lateral faces, in order to inject and measure the air pressure. The membrane is pressurized through its cavity while the front face remains at the atmospheric pressure. So, the sample was characterized under differential pressures, between 0.04 and 4 bars. Pressure regulation and measurements were carried out using both pressure controller and sensors, operating in the range of 0 to 4 bars.

Classically, these measurements are performed with a setup integrating both a laser interferometer to detect the membrane deflection, an optical system to observe interference fringes and data processing software [14,15,16,17,18]. Despite the highest resolution of the interferometric method, the experiences had shown that the interference fringes are often not well defined at very small displacements [29]. Moreover, the measurement is often focused in the centre of the membrane, preventing the observation of the deflection profile. Acquisition of load-deflection data is significantly improved using LSM. With its confocal optical system, an LSM detects in-focus reflections from a single specified focal plane along the *z*-axis [36]. This allows the extraction of the 3D deflection profile of the membrane under an applied pressure.

The deflection *h* of the membrane (at *P* = *P_atm_*) was measured before and after the chip preparation. Values of *h* are close, around 1.5 µm. So, the impact of the stress induced by the sample preparation seems to be negligible as shown in Figure 3b. The deflection profile was recorded at each stabilized pressure level. A scanning area of 4700 × 4700 µm^2^ was defined in the centre of the membrane. Thus, we obtained a mapping including the maximum deflection point in the centre and also the edges of the membrane as presented in Figure 3c.

### 2.5. Dynamic Behavior Measurements

**Figure 4:**
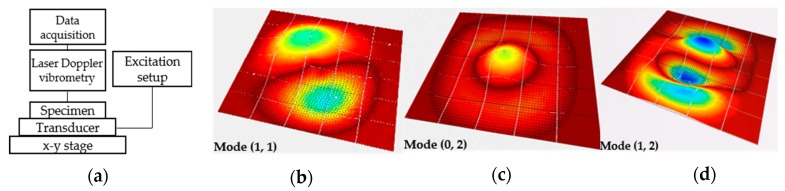
(**a**) Schematic diagram of the resonance frequency method. Vibration mode shapes measured using laser Doppler vibrometry for (**b**) (1, 1); (**c**) (0, 2); and (**d**) (1, 2) modes.

The natural frequencies of thin film can be measured by several methods. Each technique integrates mechanical excitation setup and optical system. In this study, we used a piezoelectric actuation and a laser system for measuring the film deformation amplitude. The sample was glued using silver paste onto a lead zirconate titanate (PZT) disk and excited by a periodic burst signal at a voltage range between 0.1–10 Vpp. An MSA-500 scanning laser Doppler vibrometry (LDV), from Polytec GmbH (Waldbronn, Germany), was used in order to experimentally investigate the dynamic behavior of the membrane through the identification of its resonance modes. Measurements were performed in air. The diameter of the diaphragm exceeded the aperture angle of the MSA lenses. Consequently, for one acquisition, an area of 890 × 660 µm^2^ is measured using the 20× objective. Thus, a stitching method was developed in order to scan the whole membrane surface allowing the determination of its vibrations mode shape. Among all the observed modes, we focused our attention on the first six out-of-plane vibration modes. As an example, Figure 4 shows different measured mode shapes of the 4H-SiC circular membrane.

## 3. Results and Discussion

### 3.1. Bulge Test Results

**Figure 5:**
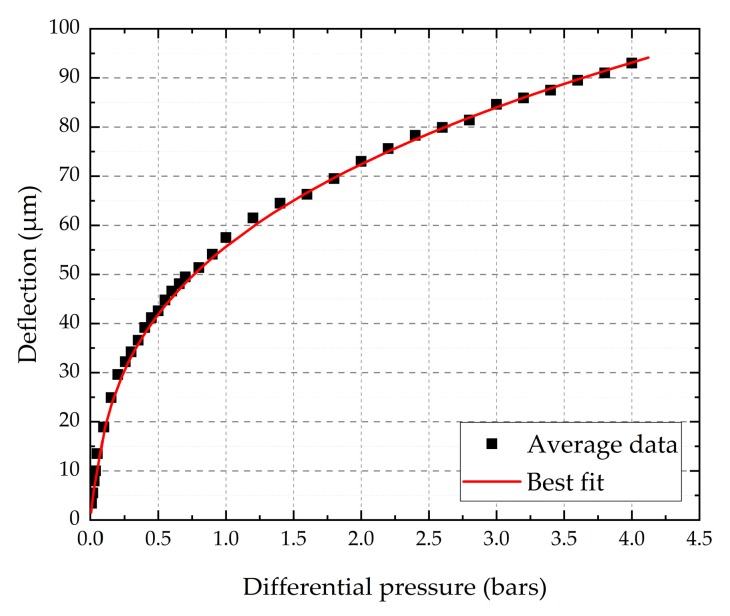
Dot line: Bulge test results for 4H-SiC diaphragm. Solid line: Theoretical fit.

As previously explained, the Young’s modulus and the residual stress can be determined using the least square fit of Equation (1). The load-deflection response at the membrane centre *h* versus *P* is shown in Figure 5.

For the calculations, we fixed the Poisson’s ratio value at 0.25, which is the most common value used in the literature. It has been pointed out by several authors that the influence of *υ* in the bulge test is negligible [34,37]. Indeed, our calculations confirm that a variation of *υ* from 0.2 to 0.3 impacts the Young’s modulus value for less than 10%. The parameters used for fitting the data are listed in Table 2.

Table 3 reports the determined values of *E* and *σ*_0_. The calculated Young’s modulus values are scattered, depending on the model used. In fact, the main difference between these models is the expression of *C_2_*. Beams was the first to report a value for this dimensionless coefficient, using the spherical cap model based on a very simple approximation of the real case. However, it can lead to an under estimation of the mechanical property determination [29,38]. Therefore, the models proposed by Pan et al. and Small et al. led to close Young’s modulus values, which seems to be normal as both models were adjusted from finite element calculations [20]. Moreover, using the numerical solution proposed by Hohlfelder, we obtained almost the same Young’s modulus value. Mitchell et al. explained that the difference in the governing equation, which results in measured values, could vary by as much as 20% [34]. In any case, the calculated Young’s modulus values are in reasonably good agreement with the published results in the literature for silicon carbide thin films. The residual stress value is determined using the linear term in Equation (1). In comparison with *E*, the stress *σ*_0_ seems to be less dependent on the model used since *C_1_* is considered as constant.

### 3.2. Vibrometry Results

**Figure 6:**
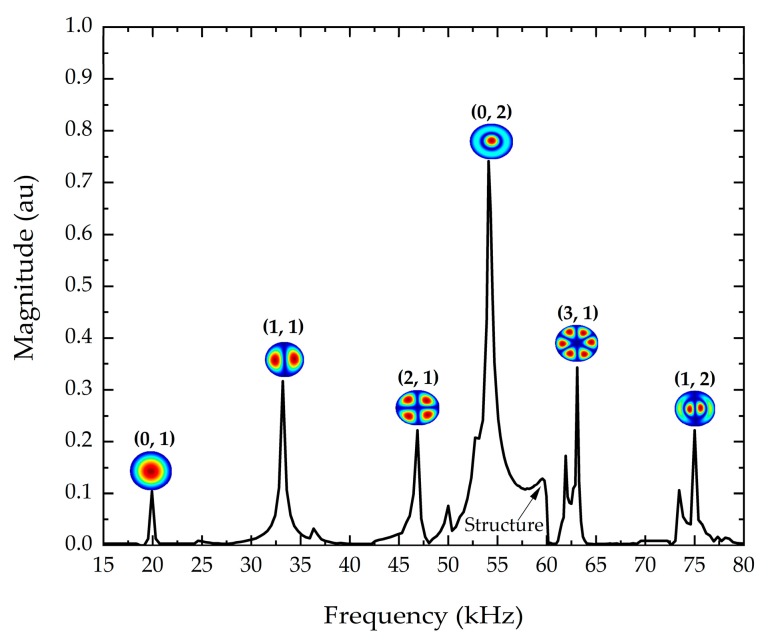
Measured spectrum of vibration of the 4H-SiC membrane associated with the corresponding mode shapes.

In this study, we focused our purpose on the observed six-first vibration modes, which are clearly identified, even if the vibration amplitudes were small. The displacement spectrum of the 4H-SiC circular membrane is shown in Figure 6. The interference frequencies due to the piezoelectric excitation were also measured. The resonance peaks for asymmetric modes (1, 1), (2, 1), (3, 1), and (1, 2) seem to be splitted with lower magnitude peaks. The fabrication process led to a geometric asymmetry of the diaphragm, as previously shown in Figure 1, resulting in the creation of non-degenerated modes in asymmetric vibration modes [39,40]. Moreover, Fartash et al. reported that the presence of an anisotropic tension, due to internal stress or tension after mounting the sample, could also cause the splitting of degenerated modes. Thus, this phenomenon could shift the peaks of asymmetric vibration modes [41].

### 3.3. Finite Element Computations

**Figure 7:**
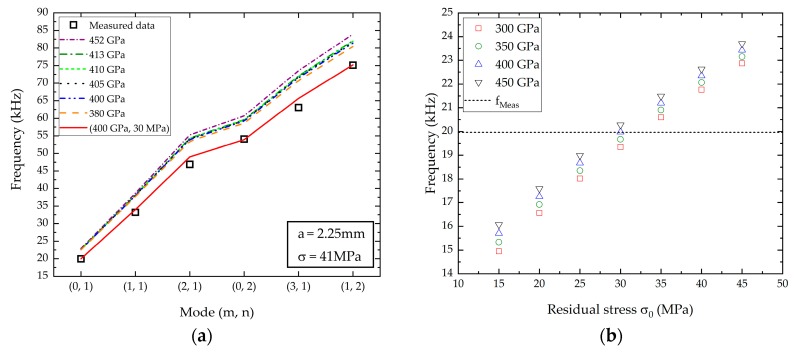
(**a**) Dashed lines: Computed resonance frequencies obtained with FEM calculations using the bulge test results. Solid line: Adjusted FEM calculations with the couple (*E*, *σ*_0_). Square symbols: Measured resonance frequencies determined with the vibrometry method; (**b**) calculated resonance frequency depending on *E* and *σ*_0_. Dot line: Measured resonance frequency for the (0, 1) mode. Symbol lines: Calculated resonance frequencies depending on the residual stress and Young’s modulus values.

The Young’s modulus and the residual stress values determined with the bulge test method were used to calculate the resonance frequencies from the implemented FEM model. Physical and geometrical parameters, already presented in Table 2, were used for the calculations. Figure 7a shows the simulated and measured resonance frequencies, depending on the vibrating mode shapes. Considering the mechanical properties extracted from the bulge test, the calculated resonance frequency values are slightly overestimated, with a difference of 15% compared to the measured values. Moreover, the Young’s modulus value seems to have a small, but not negligible, influence on the resonance frequency values. In addition, the membrane shape is not a perfect circle, as already presented in Figure 1a. Therefore, the same calculations were performed using a membrane radius of 2.35 mm, corresponding to the higher measured radius. The obtained results were very close to the preceding one, using *a* = 2.25 mm. Thus, we assumed that the radius variation has a negligible influence on the presented results. It is important to note that all the calculations were performed for a single residual stress value, i.e., 41 MPa. In order to evaluate the impact of the residual stress, we extended the calculation by changing *σ*_0_ between 15 and 45 MPa. Figure 7b presents the simulations results for the first resonance frequency. The couple (*E*, *σ*_0_) that provides the best fit with the measured resonance frequency was obtained for *E* = 400 GPa and *σ*_0_ = 30 MPa. Consequently, we used these mechanical parameters to calculate the five other resonance frequencies. We reported the results in Figure 7a. The resonance frequencies obtained by FEM simulations using *σ*_0_ = 30 MPa seem to be in good agreement with the measured resonance frequencies. Thus, it can be assumed that the residual stress value mainly governs the mechanical behavior of the membrane.

However, the residual stress value extracted from bulge test and FEM simulations are slightly different, even if the difference remains minor. This can be explained by the deflection measurements in the low-pressure range. The determination of the residual stress is allowed with the linear term of Equation (1). Thus, an error in the pressure or deflection measurements could lead to an over or under estimation of the residual stress value.

### 3.4. Etching Profile Determination

As previously explained, we considered, for our model, that the membrane was firmly clamped at its periphery. This property is essential as an undercut or overcut at the diaphragm boundary could shift the resonance frequencies, and so, could affect the accuracy of the mechanical properties determination.

**Figure 8:**
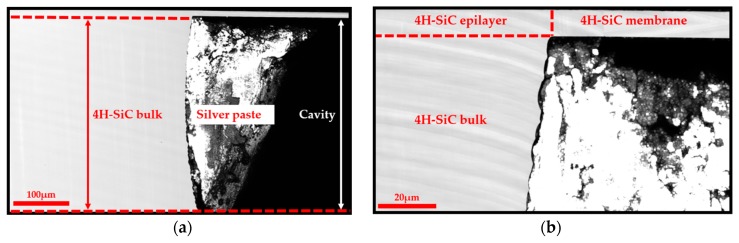
(**a**) LSM cross-section image of the etching profile; (**b**) LSM image of the membrane-undercut boundary.

Most of the time, designing freestanding diaphragms based on 3C-SiC material requires etching the Si substrate by using an anisotropic wet etchant, such as potassium hydroxide. This technique is very suitable to create thin suspended square and rectangular membranes but does not allow the achievement of vertical sidewalls. In addition, the realization of circular membrane geometry is more complex, that’s why, dry etching using plasma is recommended [39]. With this method, the Si wafer/3C-SiC epilayer interface can act as an etch-stop to define a 3C-SiC membrane. For 4H-SiC, it is much trickier. Indeed, plasma etching of 4H-SiC can also be used to define vertical sidewalls [42]. However, in this case, there is no etching-stop layer. Thus, the realization of a complete well-controlled 4H-SiC membrane is quite impossible. While, thanks to the ECE method applied in this paper, it is achievable. Nonetheless, considering this unusual method, it is mandatory to explore the etching profile. To do that, we observed the sample cross-section by means of LSM method, as shown in Figure 8.

This figure clearly highlights the anisotropic etching of the 4H-SiC wafer and the thickness homogeneity of the 4H-SiC membrane. Moreover, the assumption of a firmly clamped membrane is clearly demonstrated. It confirms that the ECE method is helpful to achieve well-defined 4H-SiC membranes.

## 4. Conclusions

The mechanical properties of a 4H-SiC circular membrane were investigated through the determination of its Young’s modulus and residual stress. Two methods were implemented and compared. The first one was based on bulge test, where the static behavior of the membrane submitted to a high external pressure was monitored. The second method was based on LDV measurements, where the dynamic mechanical response of the membrane was investigated. An inverse problem approach, based on finite element method, was implemented in order to combine the static and dynamic results, allowing the determination of the mechanical properties of the 4H-SiC circular membrane. The two methods provided very similar results since one obtained a Young’s modulus of 410 GPa and a residual stress value of 41 MPa from bulge test against 400 GPa and 30 MPa for the LDV analysis. The calculated Young’s modulus is in good agreement with literature values for SiC thin film. Moreover, the process allows the full removal of the highly doped 4H-SiC substrate and the local release of the epitaxial layer, thus realizing membranes with thickness uniformity determined by the epitaxial layer growth. Consequently, a new range of MEMS can be developed using 4H-SiC based-material.

## Figures and Tables

**Table 1:** C_1_ and C_2_ coefficients for circular membranes.

Models	C_1_	C_2_	C_2_(υ = 0.25)	Approach
Lin [28]	4.0	(7 − υ)/3	2.25	Energy minimization
Beams [27]	4.0	8/3	2.67	Spherical cap
Small et al. [29]	4.0	(8/3) × (1 − 0.241 × υ)	2.51	Finite Element Method
Pan et al. [30]	4.0	(8/3)/(1.026 + 0.233 × υ)	2.46	Finite Element Method
Hohlfelder et al. [31]	4.0	(8/3) × (1.015 − 0.247 × υ)	2.54	Number approximation
Timoshenko et al. [32]	**-**	(8/3) × 0.976/(1 + υ)	2.08	Energy minimization

**Table 2:** Parameters used for both bulge test and finite element method (FEM) calculations.

Parameters	Values
*A* (Pa/m)	3.0 × 10^8^
*B* (Pa/m^3^)	4.6 × 10^17^
Density (kg/m^3^)	3210
Poisson’s ratio	0.25
Membrane radius (µm)	2250
Membrane thickness (µm)	8.8

**Table 3:** Bulge test results depending on the models used.

Models	*E* (GPa)	*σ*_0_ (MPa)
Lin [28]	452	41
Beams et al. [27]	380	41
Small et al. [29]	405	41
Pan et al. [30]	413	41
Hohlfelder [31]	400	41
Mean value	410	41
